# Supplementary material for: Patterns of Intron Gain and Loss in Fungi
Source: PLoS Biol. 2004 Nov 30;2(12):e422. doi: 10.1371/journal.pbio.0020422 (PMC532390; doi:10.1371/journal.pbio.0020422)
Supplement: Table S1 — Also available at http://genes.mit.edu/NielsenEtAl/. (4.3 MB ZIP). [file pbio.0020422.st001.zip › NielsenEtAl/html/797.html]

AN7497.1.NCU05221.1.MG04136.1.FG02474.1


```
 CLUSTAL W (1.82) Multiple Sequence Alignments - Introns Inserted


Sequence 1: NCU05221.1	218 aa
Sequence 2: FG02474.1	197 aa
Sequence 3: MG04136.1	207 aa
Sequence 4: AN7497.1	221 aa
Alignment Length: 221 aa
Number Identitical Residues: 96 aa
Alignment Score (without introns) 4487


MG04136.1 	MASLRSSSTAAARLLRSAS------WQQTRAVVAGRRHESSLVRS-------E-KSGPAL
NCU05221.1	MSALRITTASAARMLRTSNAMMPSVMGAAQRRALSDSAEPARVPSVESARVPE-KLAKED
FG02474.1 	MAALR--SQSAARMLRS---------AAVPRVALS--AAPRRFQS---------NITSAT
AN7497.1  	MSFMRAGNIACSRVGRLVTYRNTRMLSATAFRAETSVKPSSSVADAPTTPLSESTLIQKE
          	*: :*  . :.:*: *  .   .     .   .      .  . .  ::  ..:.     

MG04136.1 	PDTDAKPANQPDYQSAFDKATS2TFSPVPKRVLDGSEPTEALPAAVLSGAPMELQARTVR
NCU05221.1	SPLATPKRNSPDYNVPIDKATS2TWTPVPKHIQNGSEEG-ILPAAVVSGAPMELQARTVR
FG02474.1 	GTITGPVANEPDYNIQADKATS2TYTPVPRSIQDGSEE--ILPAAIMSGAPMELQARTVR
AN7497.1  	TPAEAMTRHQPDYDATVDHGTS2KFSPVPKRVMDGSEPGETVPAAVLSGAPTDLQARTVR
          	        :.***:   *:.** .::***: : :***  . :***::**** :*******

MG04136.1 	2IYRPSKPATQSGTHGLRQWRMDWDVLGKGHRWENPLMGWQSSGDFMQGSHIYFKSKEEA
NCU05221.1	2IYLPSKPATQSSNSR-VLWRMDWDVLEKGHRWENELMGWQSSGDFVQGTHLTFRTKEEA
FG02474.1 	2IYQEAKPATQSGDWRGRRWRMDWDILPKGHRWENPLMGWQSSGDFMQGTHINFESKEDA
AN7497.1  	2IFRPSKPATQSGEWHSHHWRMDWDILQKGHRWENPLMGWQSSADAMQGTHLNFKTKEDA
          	 *:  :******.      ******:* ******* *******.* :**:*: *.:**:*

MG04136.1 	IAFAEKQGYEYFVQEPNERKITPKAYANNFLYSDKKLKHIRTK
NCU05221.1	IQFAEKQGYEYFVQEPNQRHFTPKAYANNFLYSPKKLKIVRTK
FG02474.1 	IHFAEKQGYEYFVQEPNSRKFAPKAYANNFLYSARKLKHIRTK
AN7497.1  	ILFAQKQGYEYFVQEPNERKFVPKAYANNFVHEPKKLKHIRTK
          	* **:************.*::.********::. :*** :***
```
